# Valsalva Maneuver during Computed Tomography for the Diagnosis of Tracheal Diverticulum: A Case Report

**DOI:** 10.7759/cureus.72726

**Published:** 2024-10-30

**Authors:** Georgia K Tsiouma, Methodios T Stavridopoulos, Anastasia A Oikonomou, Loukas D Megagiannis, Ioanna G Malamouli

**Affiliations:** 1 Otolaryngology - Head and Neck Surgery, General Hospital of Volos, Volos, GRC; 2 Radiology, General Hospital of Volos, Volos, GRC

**Keywords:** computed tomography, paratracheal air cyst, tracheal diverticulum, tracheocele, valsalva maneuver

## Abstract

Tracheal diverticula constitute a subtype of paratracheal air cysts that are characterized by a connection with the trachea through a thin neck. Patients with tracheal diverticulum rarely develop symptoms and are usually diagnosed on computed tomography (CT) performed for an unrelated indication. However, identifying the communication with the trachea on imaging may be challenging. This report presents the case of a patient who was diagnosed, incidentally, with a paratracheal air cyst on thoracic CT. The paratracheal air cyst was recognized as a tracheal diverticulum by having the patient perform the Valsalva maneuver during CT. This case suggests the use of this technique for the differential diagnosis of tracheal diverticula from other paratracheal air cysts.

## Introduction

Tracheal diverticula or tracheoceles are defined as benign projections of the tracheal wall at the level of T1-T3 vertebrae [1]. They are usually asymptomatic and most often they are detected incidentally on thoracic computed tomography (CT) [2]. The differential diagnosis from other paratracheal air cysts (PACs) is based on the communication of the cyst with the trachea through a narrow orifice [3]. This report presents the case of an asymptomatic tracheal diverticulum and proposes a method to distinguish it from other PACs using the Valsalva maneuver during a CT scan.

## Case presentation

**Figure 1:**
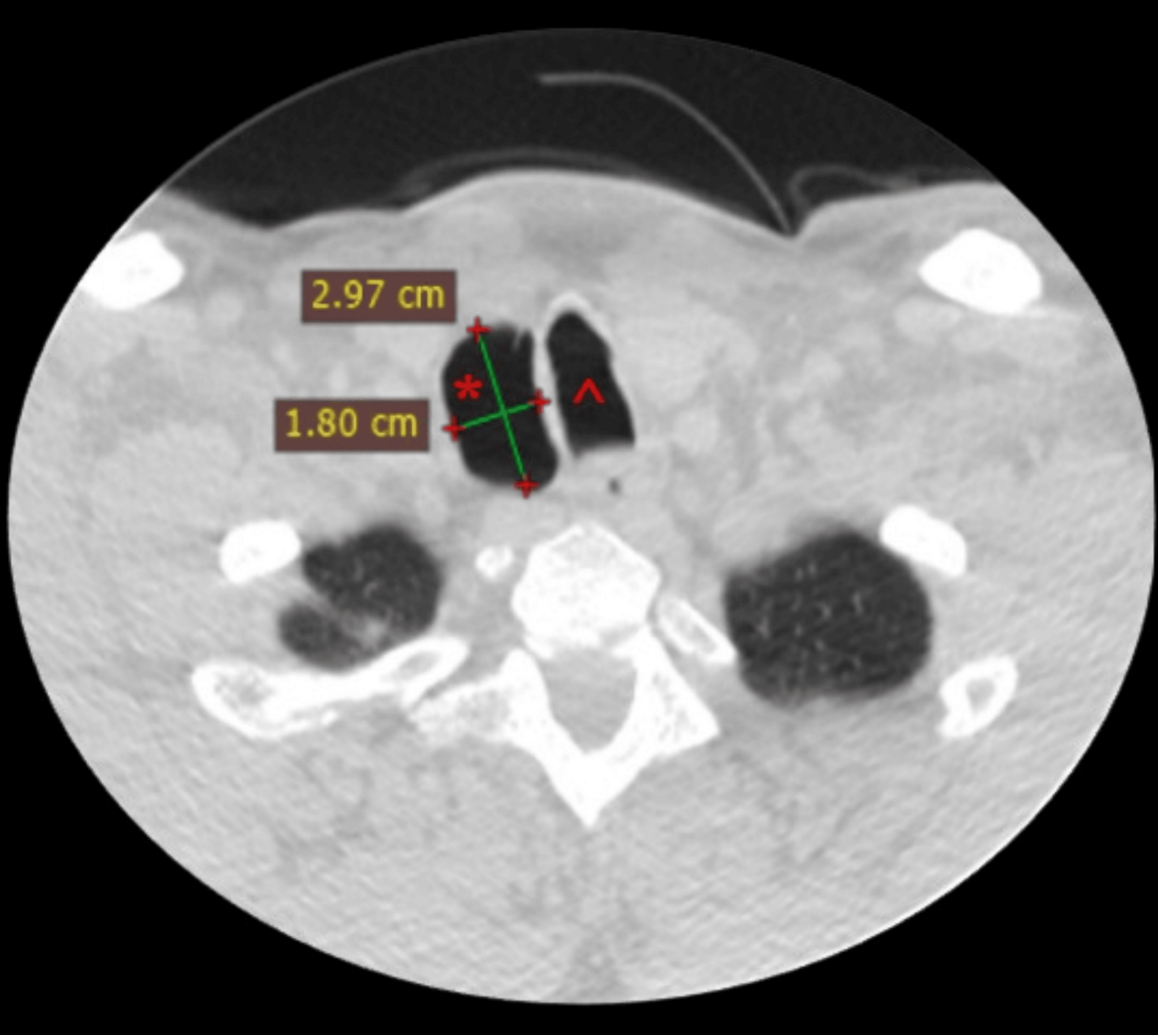
Thoracic CT scan (1.25-mm slice), axial view Right-sided tracheal diverticulum (*) with dimensions of 2.97 x 1.80 cm, without detectable connection to the trachea (^)

**Figure 2:**
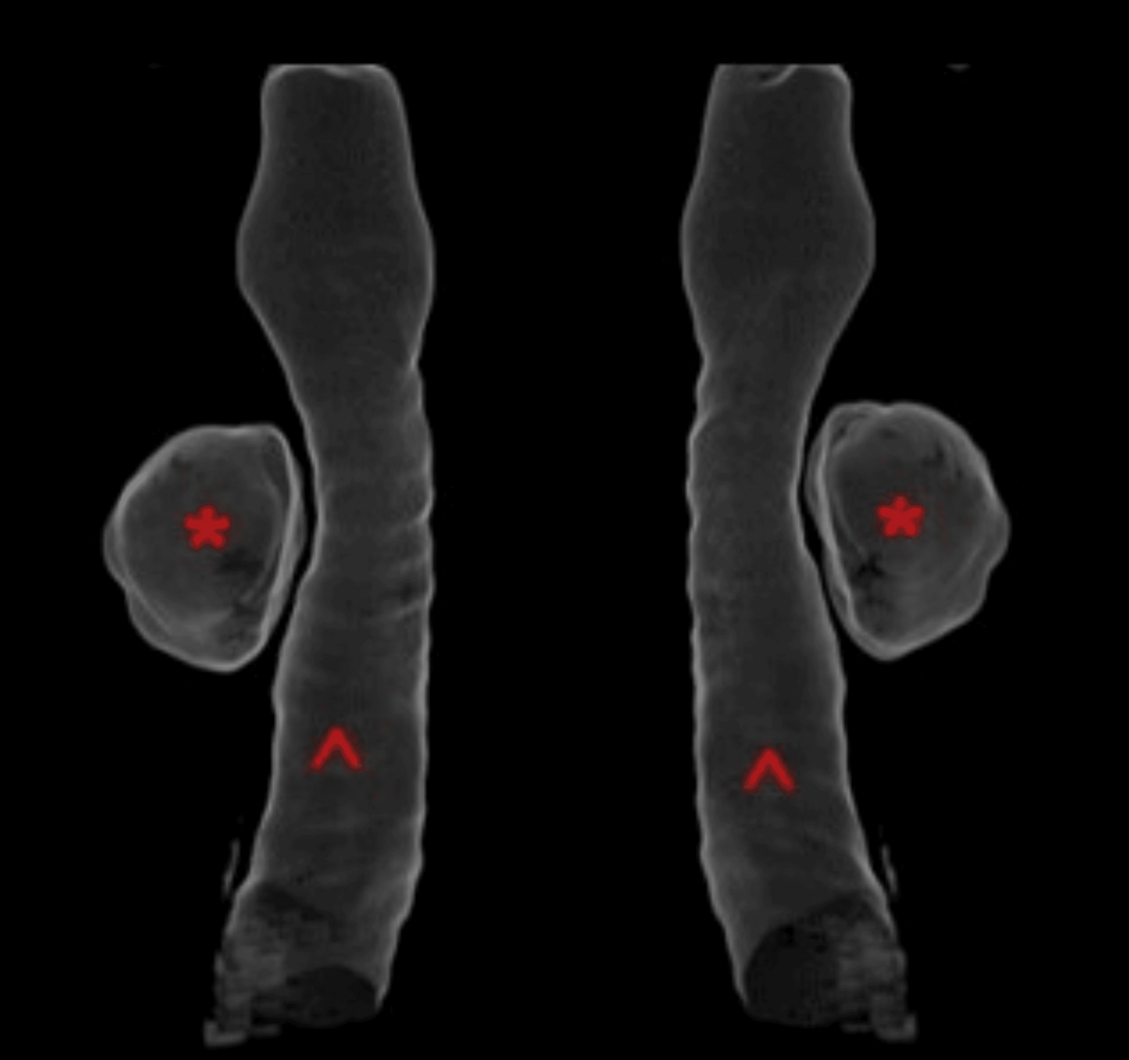
Three-dimensional reconstruction of the trachea derived from thoracic CT scan (1.25-mm slice) Right-sided tracheal diverticulum (*), without detectable connection to the trachea (^)

**Figure 3:**
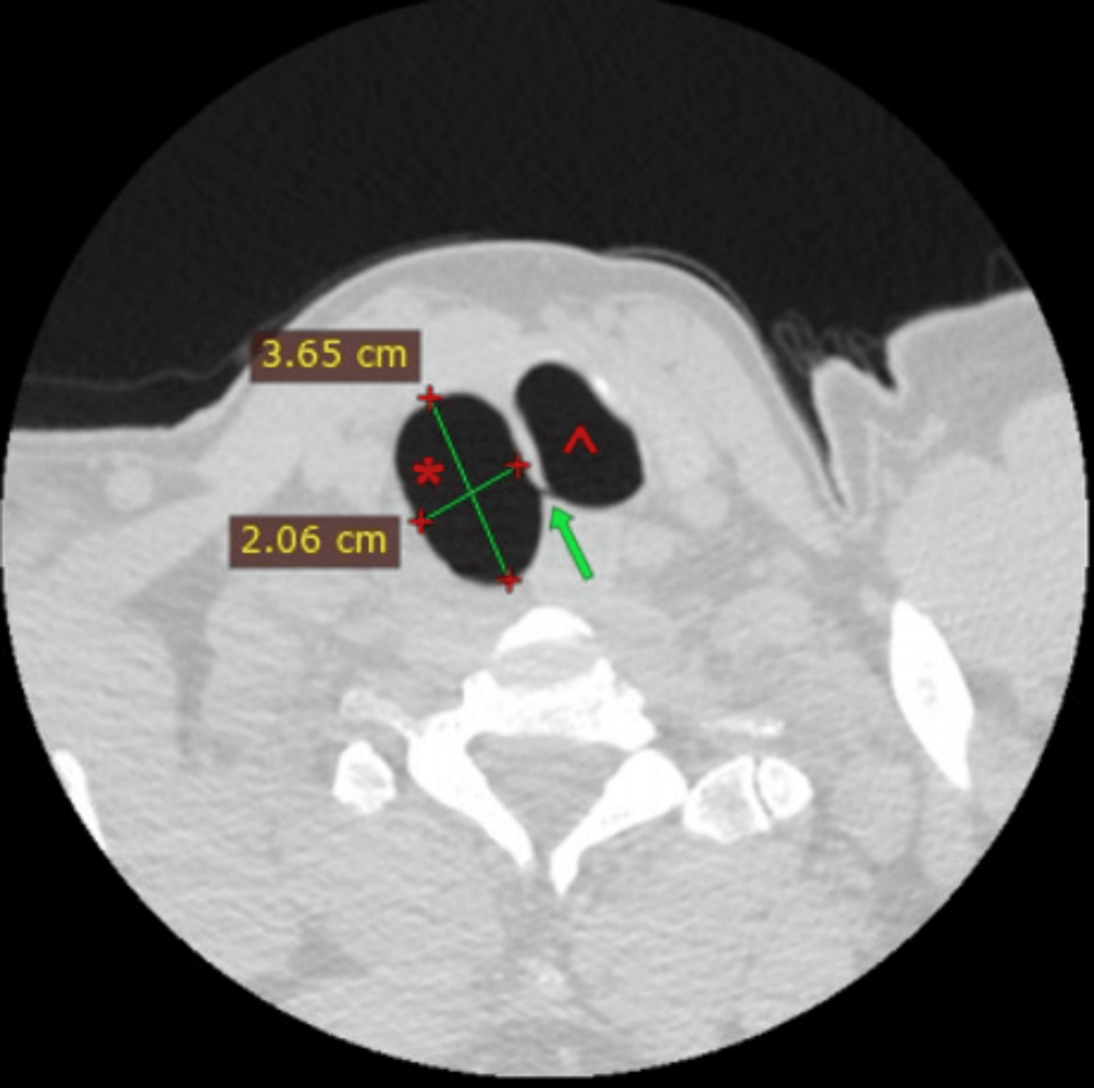
Second CT scan during Valsalva maneuver (1.25-mm slice), axial view Right-sided tracheal diverticulum (*) with dimensions of 3.65 x 2.06 cm, with detectable connection (green arrow) to the trachea (^)

**Figure 4:**
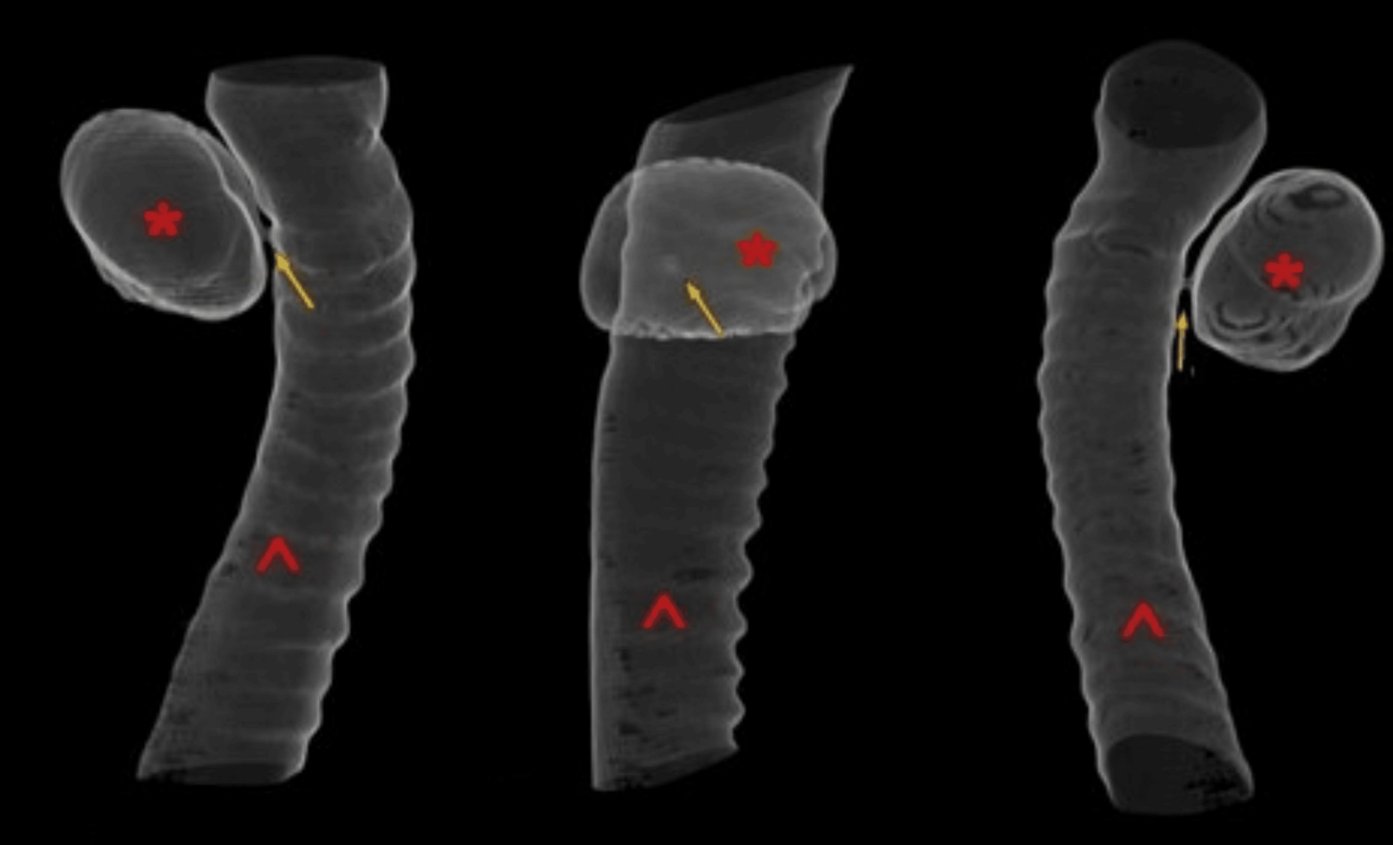
Three-dimensional reconstruction of the trachea derived from second CT scan (1.25-mm slice) during Valsalva maneuver Right-sided tracheal diverticulum (*), with detectable connection (yellow arrow) to the trachea (^)

A 55-year-old male patient was referred to the emergency department with a possible fracture of the scapula caused by a fall from a height of three meters. The patient complained only of back pain and didn’t experience dyspnea. His vitals were stable. A CT scan (1.25 mm slice thickness) was performed and besides the confirmation of a fracture of the scapula, a PAC with dimensions of 1.80 x 2.97 x 2.60 cm was identified (Figures 1, 2). In order to distinguish the type of air cyst, the patient underwent a new CT scan (1.25 mm slice thickness) while performing the Valsalva maneuver continuously for the duration of the examination, which lasted less than 10 seconds. The cyst’s volume increased in all three dimensions (2.06 x 3.65 x 2.95 cm) and a communication with the lateral tracheal wall was revealed, confirming the suspicion of tracheal diverticulum (Figures 3, 4). The patient didn’t experience any symptoms caused by the tracheal diverticulum. He was informed about his condition and maintaining regular follow-up was suggested.

## Discussion

Tracheal diverticulum is a type of PAC lined by columnar epithelium, with an incidence of 2-3.7% [1,4]. It constitutes an air cyst at the paratracheal area which is connected with the trachea through a thin neck [5]. Frequently, it is located in the right posterolateral region of the trachea at the level of the T2 vertebra, which also points to the transition of the extrathoracic to the intrathoracic trachea [6]. The mean size of tracheal diverticulum is reported as 4 mm (range 2-6 mm) and patients are usually diagnosed in the 6th decade of their life [3]. 

Tracheal diverticula are divided into acquired and congenital [4]. Congenital diverticula arise from a defect during the development of the membranous lateral tracheal wall or the tracheal cartilage [5]. Due to their origin, the presence of cartilage confirms the congenital type [7]. They are smaller, with a narrower neck than the acquired type, and may coexist with other abnormalities. They may contain mucus and typically occur 4-5 cm below the vocal folds [8]. The acquired tracheal diverticula are usually larger and are located at the entrance of the thorax. Many authors suggest that chronic respiratory diseases, such as chronic obstructive pulmonary disease (COPD), are responsible for the chronic elevated pressure at the tracheobronchial tract, which leads to the formation of tracheal diverticula [4,9]. However, this theory is not accepted universally, as there are also many authors who didn’t prove any causal relation [3,10].

Both congenital and acquired diverticula are often asymptomatic [2]. They are commonly detected incidentally on CT of the thorax or the neck. Rarely, symptoms are present which include but are not limited to chronic cough, dyspnea, dysphagia, hemoptysis, and neck pain [1]. Treatment is not necessary in asymptomatic patients, but surgical resection is often the treatment of choice for young, symptomatic patients [11,12]. Tracheal intubation in these cases may be challenging. During positive-pressure ventilation, a rupture of the diverticulum may occur, if the tracheal tube cuff is placed proximally to its neck [13]. In the literature, there are few reported cases of pneumomediastinum and subcutaneous emphysema associated with tracheal diverticulum, mainly after tracheal intubation [12,14-17]. 

Multidetector CT (MDCT) is the most effective imaging technique for tracheal diverticulum diagnosis. Magnetic resonance imaging (MRI) is a radiation-free examination that is supplementary to the diagnosis [18]. An air cyst at the paratracheal region with a connection to the trachea is characteristic of tracheal diverticulum [19]. The challenge of identifying the orifice connecting the diverticulum with the trachea can be resolved in some cases, through thinner than 1 mm slices in chest CT and multiplanar or three-dimensional (3D) reconstruction images [7,20]. MRI is useful in the detection of infected diverticula, as it can provide superior information about their wall and the extent of the inflammation [18]. Nonetheless, compared to CT, MRI has reduced capability in depicting the communication between the tracheocele and the trachea [18].

In the present case, the primary chest CT (1.25 mm slice thickness) was not able to determine a communication between the PAC and the tracheal lumen even after multiplanar reconstruction. The second CT scan (1.25 mm slice thickness) of the thoracic entrance while the patient was performing the Valsalva maneuver resulted in an elevation of the intrathoracic pressure which in turn led to the increase of the size of the tracheal diverticulum (from 1.80 x 2.97 x 2.60 cm to 2.06 x 3.65 x 2.95 cm) and allowed the observation of a narrow connection to the tracheal wall.

## Conclusions

Tracheal diverticulum is a rare type of paratracheal air cyst that is frequently asymptomatic and typically discovered incidentally during imaging procedures. Diagnosing and distinguishing tracheal diverticula from other PACs is challenging, even with advanced imaging techniques such as MDCT and multiplanar or 3D reconstruction. Accurate identification of tracheal diverticula is crucial for effective patient management, as early diagnosis may inform monitoring strategies and surgical considerations if symptoms arise. This case report proposes that differential diagnosis can be accomplished safely and effectively by utilizing the Valsalva maneuver during CT scans. While this report contributes valuable insights, it is important to recognize its limitations as a single case study, highlighting the need for future studies involving larger patient cohorts in order to validate the effectiveness of the Valsalva maneuver.

## References

[1] Tanrivermis Sayit A, Elmali M, Saglam D, Celenk C (2016). The diseases of airway-tracheal diverticulum: a review of the literature. J Thorac Dis.

[2] Kallel S, Chaabouni MA, Thabet W, Mnejja M, Ben Mahfoudh K, Charfeddine I (2022). Tracheocele: a rare entity. Iran J Otorhinolaryngol.

[3] Kurt A, Sayit AT, Ipek A, Tatar IG (2013). A multi detector computed tomography survey of tracheal diverticulum. Eurasian J Med.

[4] Goo JM, Im JG, Ahn JM (1999). Right paratracheal air cysts in the thoracic inlet: clinical and radiologic significance. AJR Am J Roentgenol.

[5] Pace M, Dapoto A, Surace A (2018). Tracheal diverticula: a retrospective analysis of patients referred for thoracic CT. Medicine (Baltimore).

[6] Kim JS, Kim AY, Yoon Y (2011). Paratracheal air cysts using low-dose screening chest computed tomography: clinical significance and imaging findings. Jpn J Radiol.

[7] Lin H, Cao Z, Ye Q (2014). Tracheal diverticulum: a case report and literature review. Am J Otolaryngol.

[8] Soto-Hurtado EJ, Peñuela-Ruíz L, Rivera-Sánchez I, Torres-Jiménez J (2006). Tracheal diverticulum: a review of the literature. Lung.

[9] Kim HY, Lee KH, Kim YJ (2017). Incidental paratracheal air cysts on thoracic CT and their association with chronic inflammatory lung disease. Biomed Res Int.

[10] Buterbaugh JE, Erly WK (2008). Paratracheal air cysts: a common finding on routine CT examinations of the cervical spine and neck that may mimic pneumomediastinum in patients with traumatic injuries. AJNR Am J Neuroradiol.

[11] Ceulemans LJ, Lerut P, De Moor S, Schildermans R, De Leyn P (2014). Recurrent laryngeal nerve paralysis by compression from a tracheal diverticulum. Ann Thorac Surg.

[12] Chakraborty A, Vaish R, Chatterjee A, Sable N, Chaukar D (2020). Tracheal diverticulum: rare presentation of known entity: a case report. A A Pract.

[13] Mitsuzawa K, Kumagai T, Uchida H, Shimizu T (2023). Positional relationships between a tracheal diverticulum and the tracheal tube under general anesthesia: a single-center observational and simulation study. BMC Anesthesiol.

[14] Allaert S, Lamont J, Kalmar AF, Vanoverschelde H (2016). Tracheal diverticulum as a cause of subcutaneous emphysema following positive-pressure ventilation. Can J Anaesth.

[15] Cherrez-Ojeda I, Felix M, Vanegas E, Mata VL (2018). Pneumomediastinum, tracheal diverticulum, and probable asthma: coincidence or possible association? A case report. Am J Case Rep.

[16] O'Leary CN, Ryan JW, Corbett G, Ridge CA (2016). Barotrauma induced tracheal diverticulum rupture: imaging findings. BMJ Case Rep.

[17] Varutti R, Rosa F, Zuccon U, Bassi F (2019). The accidental discovery of a tracheal diverticulum. Intensive Care Med.

[18] Zhang Y, Tan Y, Chen J, Fang C (2022). The role of MRI in the diagnosis and management of tracheal diverticulum. BMC Med Imaging.

[19] Abdul-Hadi Martinez S, Ruiz-Mojica CA, Rivera-Rivera G, Perez-Ortiz G, Pascual Marrero J, Pacheco Lopez P (2023). Incidental tracheocele as an unusual presentation of pneumomediastinum in the trauma setting. Am J Case Rep.

[20] Polat AV, Elmali M, Aydin R, Ozbay A, Celenk C, Murat N (2014). Paratracheal air cysts: prevalence and correlation with lung diseases using multi-detector CT. J Med Imaging Radiat Oncol.

